# A new method for facilitating ultrasound-guided in-plane cannulation of the subclavian vein: a randomized clinical trial

**DOI:** 10.1038/s41598-021-88798-0

**Published:** 2021-05-05

**Authors:** Qingxiang Mao, Haitao He, Yuangang Lu, Yi Hu, Zhen Wang, Maoxiang Gan, Liyong Chen, Hong Yan

**Affiliations:** 1 Department of Anesthesiology, Daping Hospital, Army Medical University, 10 ChangjiangZhilu, Yuzhong District, Chongqing, 400042 China; 2 Department of Maxillofacial and Head and Neck Surgery, Daping Hospital, Army Medical University, Chongqing, 400042 China; 3 Department of Plastic and Cosmetic Surgery, Daping Hospital, Army Medical University, Chongqing, 400042 China

**Keywords:** Comorbidities, Ultrasonography

## Abstract

The objective of this study was to propose a new method for facilitating needle-beam alignment ultrasound-guided in-plane catheterization of the subclavian vein (SCV). Three hundred patients were recruited, and ultrasound examination of the SCV was performed. Then, the patients were divided into two groups and SCV catheterization was performed: ultrasound-guided catheterization with the aiming method (group A) and ultrasound-guided catheterization with needle guide (group NG). The success rate, insertion time, number of skin breaks, number of needle redirections, needle visibility and rate of mechanical complications were documented and compared for each procedure. To depict the optimum long-axis view of the SCV, there was a 30° ± 7.3° angle (rotation) between the long axis of the ultrasound probe and the clavicle, while there was a 39° ± 7.4° angle (tilt) between the ultrasound beam plane and the right chest wall. The aiming method was associated with fewer skin breaks [(mean (IQR): 1 (1–1) times *vs* 1 (1–2) times, *P* = 0.009], a shorter time to cannulation [(mean (IQR): 39 (32–48.5) s *vs* 48 (44–54.8) s, *P* = 0.000] and more needle redirections [(mean (IQR): 0 (0–1) *vs* 0 (0–0), *P* = 0.000]. There were no differences between group A and group NG in the overall success rate, first puncture success rate, needle visibility or mechanical complication rate. In conclusion, during ultrasound-guided in-plane catheterization of the SCV, the aiming method provides comparable needle-beam alignment with a lower cannulation time than the needle guide technique.

## Introduction

Catheterization of the subclavian vein (SCV) is associated with fewer cases of thrombosis and infectious complications and improved patient comfort^1–3^ when compared to the alternative central venous access. Unfortunately, mechanical complications of SCV catheterization using landmark (or “blind”) techniques also occur more frequently^4,5^. The ultrasound-guided SCV catheterization technique has been explored and demonstrated to improve the success rate and reduce the complication rate, particularly when using the real-time in-plane method^6,7^.

Experiences in ultrasound-guided regional anesthesia indicate that a failure to align the needle and ultrasound beam imposes the risk of accidentally damaging structures not visible on the ultrasound screen^8,9^. A needle guide can be placed on the ultrasound probe and this approach has been found to facilitate alignment of the ultrasound beam and needle plane in both human and phantom studies^10,11^ compared with the freehand technique. Unfortunately, the drawbacks of needle guides, including limited degrees of needle freedom and increased costs of the procedures for patients, hinder the acceptance of this guidance technique^10,12,13^.

In this study, we introduced a new aiming method (freehand) to facilitate needle-beam alignment, since it is similar to sight alignment in shooting sports. We hypothesized that this aiming method has comparable alignment performance with the needle guide technique.

## Methods and materials

### Study design

The study protocol was approved by the Ethics Committee of Daping Hospital (2018–45). The trial was registered prior to patient enrollment at clinicaltrials.gov (NCT03778437, date of registration: December 3rd, 2018, principal investigator: Qingxiang Mao)^14^. The prospective study was conducted in the operation room from February 2019 to December 2019. Written informed consent was obtained from all subjects participating in the trial. The study was performed in accordance with all relevant national and international guidelines including the Declaration of Helsinki.

### Study population

**Figure 1:**
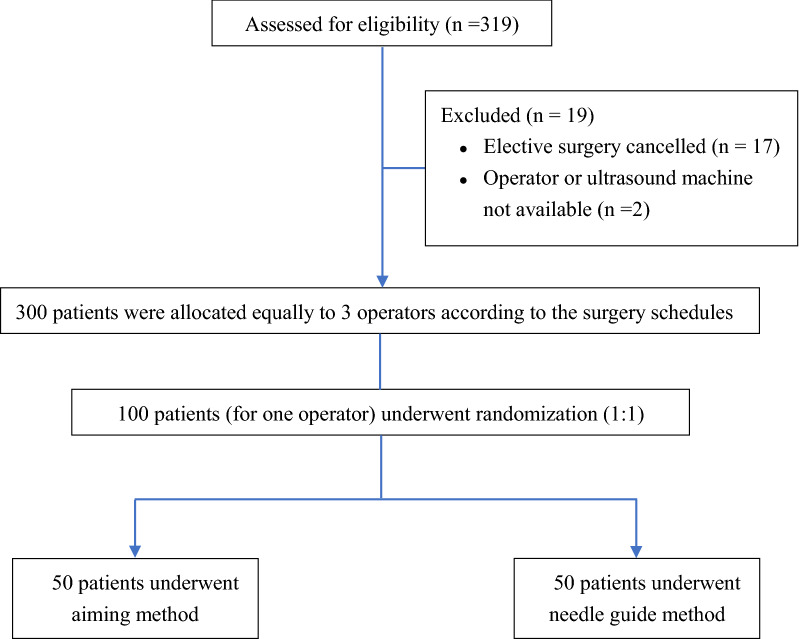
The CONSORT flow diagram of the study. Flow diagram of study subjects receiving an ultrasound-guided in-plane cannulation of the subclavian vein by aiming method or needle guidance.

The inclusion criteria were adult patients presenting for elective brain surgery and requiring SCV cannulation (Fig. 1). Patients were not recruited if there was any anatomical abnormality, SCV thrombosis or any prior history of coagulation abnormality. All patients provided written informed consent and were randomly allocated to two groups, ultrasound-guided catheterization with the aiming method (group A) and ultrasound-guided catheterization plus needle guide (group NG), by a computerized randomization table. During SCV cannulation, all patients were anesthetized and placed in the Trendelenburg position.

### SCV techniques and data collection

**Figure 2:**
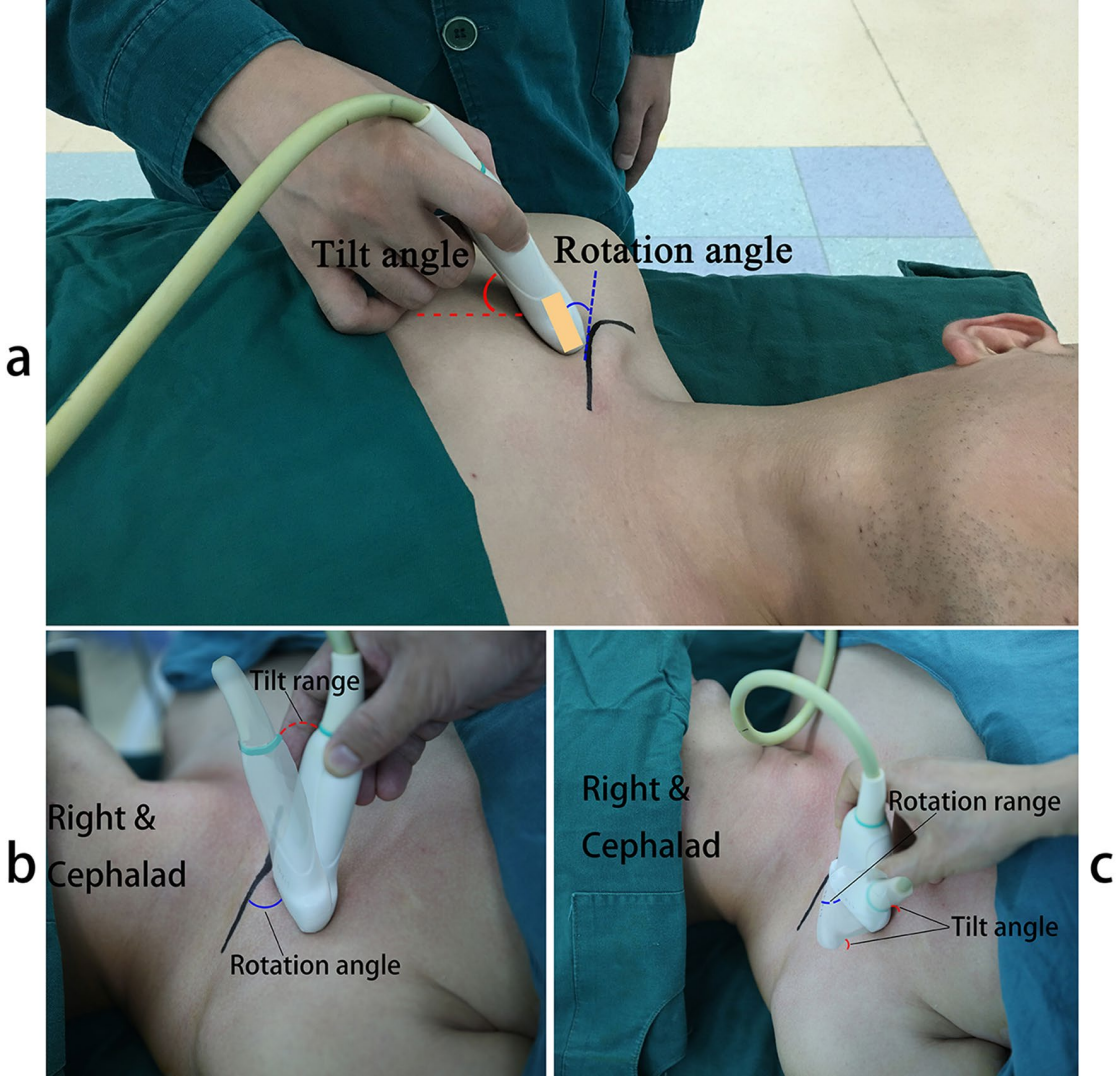
The rotation and tilt angle of ultrasound probe and the acceptable range of probe manipulation. (**a**) The rotation (green) angle between long axis of probe and clavicle; and the tilt (red) angle between ultrasonic beam plane and right chest-wall plane. (**b**) The acceptable range of probe tilt before the clear visualization of SCV disappeared. The optimal rotation angle was fixed. (**c**) The acceptable range of probe rotation before the clear visualization of SCV disappeared. The optimal tilt angle was fixed.

All patients underwent an SCV ultrasound examination. The examination started with a sagittal view depicting the right SCV on the short axis, and then the probe was rotated and tilted to display the SCV with the maximum diameter on the long-axis view. At the expected insertion site, the diameter of the SCV was recorded on both the short-axis and the long-axis views. The (rotation) angle between the long axis of the probe and the clavicle and the (tilt) angle between the ultrasound beam plane and the right chest wall plane were recorded (Fig. 2a). For the rotation angle, we first marked the point of intersection of the probe and the clavicle, then drew two lines that started from the intersection and were parallel to the long axis of the probe footprint and the clavicle on the surface of the chest; finally, the rotation angle was measured with a semicircular protractor. For the title angle, the flat edge of the protractor was placed on the chest, and the center mark was overlaid with the distal side of the probe; finally, the degree marking where the ultrasound beam plane crossed the curved edge of the protractor was read.

During each ultrasound examination, after the optimal tilt and rotation angle were determined, the operator tilted the probe left or right (while keeping the optimal rotation angle fixed) until clear visualization of the SCV disappeared, to determine the acceptable range of probe tilt (Fig. 2b). Then the operator rotated the probe clockwise or counterclockwise (while keeping the optimal tilt angle fixed) to determine the acceptable range of probe rotation (Fig. 2c).

**Figure 3:**
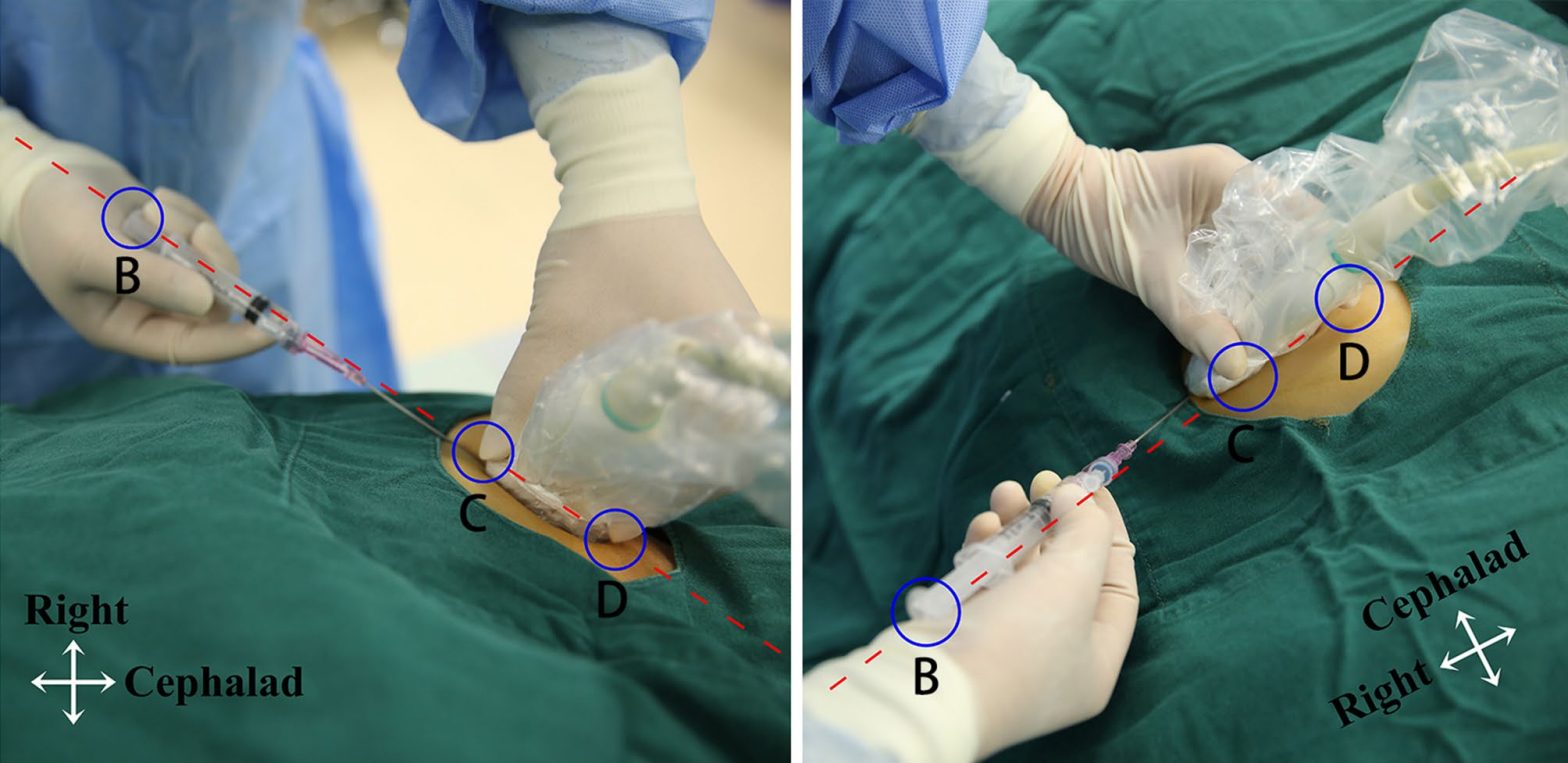
Position of operator, probe, and needle in aiming method. One point on the syringe (preferably the flat end of plunger (point B), green circle) and two points on the on the ultrasonic beam plane (preferably the thumb finger (point C) and the index finger tip (point D) on the ultrasound probe, green circles) were assigned as reference points. Aligning those three points in a line (red lines) during needle insertion will help the alignment. The operator needs to move the standing position to keep the long axis of ultrasound probe parallel to the operator’s midsagittal plane.

The aiming method is generated from a simple geometrical postulate: if two points lie in a plane, then the line joining them lies in that plane. The key points of this method include the following: I. The puncture site where the needle is inserted into the skin must be exactly underneath the ultrasound probe, which ensures one point (point A) of the needle on the ultrasound beam. II. Three points, including 1 point on the syringe (as point B, preferably the flat end of plunger) and 2 points on the ultrasound probe (as point C and D, preferably the slightly exposed thumb (C) and index finger (D) tips which were holding the probe and on the ultrasound beam plane), were assigned as reference points. III. When aligning those three reference points (B, C, D) in a line (Fig. 3), points A and B and the needle (assumed as a line) will theoretically join in the plane of the ultrasound beam. IV. The operator needs to adjust his/her standing position to keep the long axis of the ultrasound probe parallel to his/her midsagittal plane, and tilt his/her head to facilitate alignment. V. Once the vessel is clearly viewed on the ultrasound screen, the probe should not be manipulated. In order to get the clear view of the needle simultaneously, the operator should always swing the syringe (to align the flat end of plunger with two reference points on probe) while keeping the insertion point right underneath the probe (see Supplementary Video S1 online).

Three right-handed anesthesiologists who had performed more than 40 cases of SCV catheterization with the landmark (blind) method were recruited to perform SCV catheterization in real patients. Prior to this study, they were provided 60 min of standardized instructions on how to obtain the longitudinal views of the SCV. Each operator performed SCV catheterization with two different methods in patients from group A and group NG.

For patients in group A, SCV catheterization was performed with the aiming method when the SCV on the long-axis view was displayed on the middle of the screen. For patients in group NG, SCV catheterization was also performed under ultrasound guidance. During needle insertion, the needle was secured in the needle guidance device, which maintained the alignment of the needle and ultrasound beam. The primary outcome of this study was the success of the procedure which was defined as the guide wire positioned into the SCV and confirmed by ultrasound. The second outcome measure was the number of skin breaks. If three attempts (skin breaks) were unsuccessful, SCV catheterization was considered to have failed and the internal jugular vein was chosen for cannulation. All attempts in the two groups were recorded by a video camera and analyzed by an attending anesthesiologist who was blinded to the study objectives. At the end of the procedure, the presence of complications was checked by ultrasound as previously described^15^.

### Data analysis

The performance outcome data are reported as the median with the mean ± SD. Statistical analysis was conducted by the Mann–Whitney U test to compare the performance outcomes. The chi-squared test was used to compare categorical variables. The demographic characteristics of the study participants are reported as the mean ± SD. The sample size was calculated assuming that the time to cannulation in group A was less than that in group NG. Almost 150 patients per group were required to detect a 15% decrease in the time to cannulation in group A (alpha = 0.025, power = 0.8, superiority margin = -4). The level of significance was set at *P* < 0.05.

## Results

A total of 300 patients were enrolled in this study. There were no significant differences in patient characteristics between group A and group NG (Table 1).

**Table 1:** Performance of resident with aiming methods in human study. Data were described as mean ± standard variation or proportion.

Variables	Aiming method (n = 150)	Needle guide (n = 150)	*P* value
Overall success rate (%)	96	97.3	0.75
Skin breaks (times)	1.1 ± 0.33 ^a^	1.4 ± 0.76	0.009
Needle redirections (times)	0.33 ± 0.79 ^a^	0.03 ± 0.18	0.000
First puncture success rate (%)	77.3	74.7	0.589
Time to cannulation (s)	43.1 ± 13.2 ^a^	50.9 ± 10.9	0.000
Needle visibility	0.77 ± 0.25	0.79 ± 0.12	0.232
**Complications**			
Number of arterial punctures (cases)	0	1	1.0
Number of pneumothorax (cases)	1	0	1.0

^a^*P* < 0.05 compared with the needle guide group (group NG).

Needle visibility: the fraction of time the needle tip was visualized during the insertion.

Time to cannulation: The time from the skin was punctured to guide-wire insertion.

First puncture success rate: The percentages of cases who were catheterized in first puncture try without needle redirections.

### Optimal position of the ultrasound probe to depict the SCV

When the ultrasound probe was placed inferior to the lateral half of the right clavicle and the SCV was depicted with a clear picture of the venous wall and with the maximum diameter on the long-axis view, the (rotation) angle between the long axis of the probe and the clavicle was 30° ± 7.3° (n = 300), which made the probe point to the sternal notch, and the (tilt) angle between the ultrasound beam plane and the right chest-wall plane was 39° ± 7.4° (n = 300).

In the long-axis view, the maximum diameter of the SCV at the expected insertion site of the vessel was 1.09 ± 0.17 cm (n = 50), 1.09 ± 0.15 cm (n = 50) and 1.08 ± 0.16 cm (n = 50), as measured separately by each operator, and was 1.09 ± 0.16 cm (n = 300) as a whole. In the short-axis view, the maximum diameter of the SCV was 1.07 ± 0.15 cm (n = 50), 1.06 ± 0.15 cm (n = 50) and 1.04 ± 0.14 cm (n = 50), as measured separately by each operator, and was 1.05 ± 0.15 cm (n = 300) as a whole. There was no significant difference in the maximum diameter of the SCV between the long-axis and the short-axis views. There was also no difference in the measurement between individual operators. This indicates that this long-axis scanning method can depict the central part of the SCV in most patients.

When the optimal rotation angle was determined and fixed, we changed the tilt angle of the probe and found that the acceptable range of probe tilt was 18° ± 4.8° (n = 300) before clear visualization of the SCV wall disappeared. In the same way, when the probe tilt was fixed at the optimal tilt angle, we changed the rotation angle and found that the range of probe rotation was 5° ± 1.3° (n = 300). These narrow ranges of probe movement suggest that it is unwise to manipulate the probe for the purpose of needle-beam alignment during SCV insertion.

### Aiming method facilitates SCV catheterization

Three experienced residents were recruited to perform SCV insertion in patients with the aiming method (n = 150) and the needle guide technique (n = 150). When compared with the needle guide method (group NG), the aiming method (group A) was associated with fewer skin breaks [(mean (IQR) 1 (1–1) times *vs* 1 (1–2) times, *P* = 0.009], a shorter time to cannulation [(mean (IQR) 39 (32–48.5) s *vs* 48 (44–54.8) s, *P* = 0.000], and more needle redirections [(mean (IQR) 0 (0–1) *vs* 0 (0–0), *P* = 0.000] (Table 2). There was no difference between the aiming group and the needle guide group in the overall success rate (96% *vs* 97.3%, *P* = 0.75), first puncture success rate (77.3% *vs* 74.7%, *P* = 0.589) or needle visibility [(mean (IQR) 0.77 (0.66–0.88) *vs* 0.79 (0.73–0.86), *P* = 0.232] (Table 2). One arterial puncture occurred in group NG, and one case of pneumothorax occurred in group A. No significant differences were observed between the two groups (Table 2).

**Table 2:** Demographic variable of participating patients.

Patients characteristic variables	Aiming method (n = 150)	Needle guide (n = 150)
Sex (male/female)	85/65	74/76
Age (years)	52 ± 14.4	51 ± 11.6
Weight (kg)	63 ± 11.4	61 ± 12.9
Height (cm)	158 ± 10.2	159 ± 11.7
BMI (kg/m^2^)	24.9 ± 2.8	23.8 ± 3.1
**ASA physical status**		
2	77 (51.3%)	67 (44.7%)
3	73 (48.7%)	83 (55.3%)

## Discussion

A variety of technologies have been developed to facilitate the alignment of the needle and ultrasound beam^12^. Extra and expensive hardware or other limitations hinder the acceptance of these guidance techniques^11^. In this study, we proposed a new aiming method that may facilitate the alignment of the needle with the ultrasound beam, and we found that the aiming method provided comparable needle-beam alignment with a shorter cannulation time than the needle guide technique during ultrasound-guided in-plane catheterization of the SCV.

Identifying the anatomy of the insertion site and the location of the SCV are key to successful catheterization. Previous studies and reviews have recommended identifying the SCV and its anatomical relation to the artery using a short-axis view first and then rotating the probe to obtain a long-axis view of the SCV^11,16^. According to a recent anatomical study, if the probe is placed inferior to the lateral half of the right clavicle and tilted cephalad, the possibility of the SCV appearing superior to the subclavian artery on the screen is 94%, and the possibility of the SCV firstly appearing on the screen is 96%^17^. In this study, we found that when depicting the SCV with the maximum diameter on the long-axis view, there was a 30° ± 7.3° angle between the long axis of the probe and the middle third of the right clavicle, and a 39° ± 7.4° angle between the ultrasound beam plane and the chest-wall plane. The diameter of the SCV on the long-axis view was the same as that on the short-axis view, which means that this long-axis scanning method can depict the entire central axis instead of part of the central axis of the SCV. According to these results, we developed a quick method to identify the SCV: 1. The probe was placed inferior to the lateral half of the right clavicle. 2. The medial side of the probe was fixed to the middle of the clavicle and the probe was rotated to make its long axis point to the sternal notch (approximately 30° of rotation). 3. The probe was tilted cephalad gradually (approximately 40° between the chest wall and the ultrasound beam), and the long-axis view of the SCV, which is superior, larger in diameter, not pulsatile and compressible compared with the artery, was obtained (see Supplementary Video S1 online).

During ultrasound-guided catheterization, it is rational to depict the SCV with the maximum diameter. Doing so allows a great range of deviation of the needle tip from the ultrasound beam that will reduce the chances of missing the vessel during needle insertion. To depict the SCV with its maximum diameter, the ultrasound probe was tilted from the skin surface and rotated from the clavicle. It is recommended that the operator tilt his/her head or body to keep the line of sight parallel to the ultrasound beam plane^13,18^. Therefore, we propose this aiming method to remind the operator to move his/her standing position (eliminating the rotation angle) and tilting their body or head (eliminating the tilt angle) to keep the line of sight parallel to the ultrasound beam plane.

In ultrasound-guided procedures, it is common to manipulate (slide, tilt, and rotate) the probe to regain the lost view of the needle tip^19^. In ultrasound-guided in-plane SCV catheterization, however, manipulation of the probe is not helpful because when depicting the SCV with its maximum diameter, the range of probe tilt and rotation are limited to 18° and 5°. Slight movement of the probe will lead to loss of the optimum image of the SCV. The inserted needle will limit any potential adjustment of the needle guide or probe and result in more puncture attempts; this partially explains the higher skin breaks and longer time to cannulation in group NG in our study. It should always be kept in mind that a clear image of the SCV is more helpful than a clear trajectory of the needle tip. In the case of pneumothorax in group A, the operator manipulated the probe to track the needle and advanced the needle without a sonographic depiction of the SCV.

Maecken and colleagues found that the needle guide technique leads to better needle-beam alignment than the simple freehand method^12^. In this study, we found a similar needle-beam alignment performance with the aiming method (freehand in nature) and the needle guide method. The different findings might be a result of the differences in the operators’ previous experiences with ultrasound-guided procedures and usage of the aiming method in our study.

It is recommended to utilize an in-plane technique instead of an out-of-plane technique for vascular access because the entire needle and the depth of the needle tip can be visualized on ultrasound images^7,20^. However, in a recent study, Vezzani et al. stated that the out-of-plane procedure for ultrasound-guided SCV catheterization offers advantages over the in-plane procedure^21^. In this study, the probe was almost parallel to the clavicle during the in-plane procedure which is different from our findings. The scanning method of the SCV used in Vezzani’s study may not depict the SCV clearly or with the maximum diameter, increasing the chance of the needle tip missing the SCV and leading to a decreased success rate^21^. However, further study is still needed to obtain a definite conclusion.

The results of our study were obtained by just three experienced operators, and thus cannot be directly extrapolated to physicians with varying levels of experience. These operators were also not blinded to the hypotheses of the study. This lack of blinding may have led to unconscious bias and impacted the results of our study.

In conclusion, during ultrasound-guided in-plane SCV catheterization the aiming method was associated with a comparable success rate, fewer skin breaks and a shorter cannulation time, but a higher needle redirection, when compared with the needle guide technique. The aiming method may be an alternative method to the needle guide technique.

## Supplementary Information

Supplementary Video 1.
